# New determinants of olfactory habituation

**DOI:** 10.1038/srep41047

**Published:** 2017-01-25

**Authors:** Charlotte Sinding, François Valadier, Viviana Al-Hassani, Gilles Feron, Anne Tromelin, Ioannis Kontaris, Thomas Hummel

**Affiliations:** 1 Smell & Taste Clinic, Department of Otorhinolaryngology, TU Dresden, Dresden, Germany; 2 Open Value, Paris, France; 3 Centre des Sciences du Goût et de l’Alimentation, CNRS, UMR 6265, INRA, UMR 1324, Université de Bourgogne, Dijon, France; 4 Givaudan UK Ltd, Ashford, UK

## Abstract

Habituation is a filter that optimizes the processing of information by our brain in all sensory modalities. It results in an unconscious reduced responsiveness to continuous or repetitive stimulation. In olfaction, the main question is whether habituation works the same way for any odorant or whether we habituate differently to each odorant? In particular, whether chemical, physical or perceptual cues can limit or increase habituation. To test this, the odour intensity of 32 odorants differing in physicochemical characteristics was rated by 58 participants continuously during 120s. Each odorant was delivered at a constant concentration. Results showed odorants differed significantly in habituation, highlighting the multifactoriality of habituation. Additionally habituation was predicted from 15 physico-chemical and perceptual characteristics of the odorants. The analysis highlighted the importance of trigeminality which is highly correlated to intensity and pleasantness. The vapour pressure, the molecular weight, the Odor Activity Value (OAV) and the number of double bonds mostly contributed to the modulation of habituation. Moreover, length of the carbon chain, number of conformers and hydrophobicity contributed to a lesser extent to the modulation of habituation. These results highlight new principles involved in the fundamental process of habituation, notably trigeminality and the physicochemical characteristics associated.

Habituation is the fundamental mechanism that allows filtering of the constant stream of information that reaches our sensory receptors. In doing so, it frees up processing resources for attentive processes. Underlying mechanisms are likely optimized to allow habituation and recovery in fast changing environments. Although habituation occurs in all sensory modalities, some of its most noticeable occurrences take place in the olfactory system. For example, it is common to experience a rapid fading of the smell of sweat when entering a crowded place. A lack of olfactory habituation may lead to severe and debilitating chemical intolerance as shown in patients who reported impossibility to study or work in a room where certain odors are present^1^. The researchers investigating this condition found that patients did not exhibit the typical decrease in intensity over time and that the patients’ brain processed the odors differently than the controls.

Habituation may result from at least two mechanisms, peripheral adaptation at the level of the olfactory receptors or central adaptation at the level of the central nervous system. In the current investigation we used the term habituation for the perceptual decrement of odor intensity (sensory response) as proposed by the Thompson group^2^, and we used the term adaptation when addressing the underlying mechanisms. Although there is no proof yet of short term olfactory receptor adaptation in humans^3^, adaptation mechanisms could take place at the level of the olfactory receptor, potentially through the feedback modulation caused by Ca^2+^ entry into the neuron^4^,^5^. Furthermore, adaptation may occur in the central nervous system likely through inhibitory processes. Mechanisms of central adaptation have been shown in rats where single neurons in the anterior piriform cortex selectively adapt despite relatively maintained input from the olfactory bulb^6^,^7^,^8^,^9^. In humans, fMRI data revealed that the orbitofrontal cortex (integrative area for olfaction) decreased the activity of the piriform cortex (primary processing area after the olfactory bulb)^10^.

Without habituation our brain would be overstimulated and would not be able to compute any other information. This process is thus fundamental but also entirely unconscious, which raises important questions. First, is habituation similar for any odorant perceived in a similar context without any ecologically relevant task, and if not, what molecular of perceptual information contribute to the specific decrease of the input of one odorant or another? Rankin and her 15 collaborators^11^ recently revisited the cornerstone studies of the Thompson group^2^,^12^,^13^, by redefining habituation and proposing a revised version of the 10 characteristics of habituation. Among all the characteristics, only one relates to the intrinsic properties of the odor perceived. “Within a stimulus modality, the less intense the stimulus, the more rapid and/or more pronounced the behavioral response decrement.”. To best of our knowledge, Stone *et al*.^14^ are the only ones who studied how intrinsic molecular properties of odorants could modulate habituation. They measured the modulation of the absolute detection threshold after 100 s continuous exposure to 7 odorants varying in different physicochemical properties. They found that vapour pressure was correlated to habituation; however the effect appeared only when one odorant was suppressed. Moreover, the experiment would have benefited from a higher number of subjects, as the results were based on 3 to 5 subjects only. We hence decided to push this methodology further and we tested the habituation to 32 odorant molecules varying in physicochemical properties (Table 1), during continuous stimulations of 120 s in 58 subjects. Subjects evaluated continuously odorant intensity with a non-declarative method designed to highlight any change in the perceived odor intensity. The first part of the analysis addressed whether habituation is homogeneous among odorants, while the second part aimed at determining the relative contribution of variables in a large set of physico-chemical and perceptual variables to habituation.

## Results

### Clustering analysis

**Figure 1:**
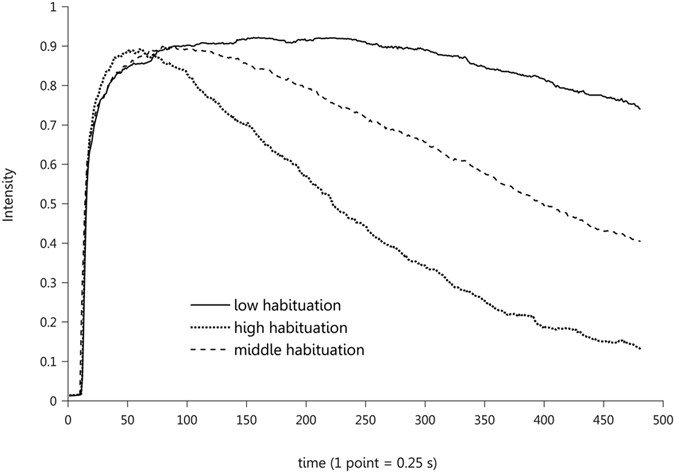
Centroid curves resulting from the clustering of the data into 3 clusters. They present the intensity perceived by the subjects during 120 s. The continuous black line represents a low habituation, the dashed line represents a mid habituation and the dotted line represents a high habituation.

As *a priori* no biologically relevant parameters could be extracted from the curves; a no *a priori* method of kmeans clustering was used. The choice of three clusters permitted to form the most homogeneous groups of data (Supplementary Figure 1). The three centroid curves differed in terms of habituation with one showing a high habituation, a second one, showing a mid habituation and the last one a low habituation (Fig. 1). This clustering separate the data according to habituation, which not only confirms the variability of habituation but also validates the method of evaluation.

### Differences of habituation between odorants

**Figure 2:**
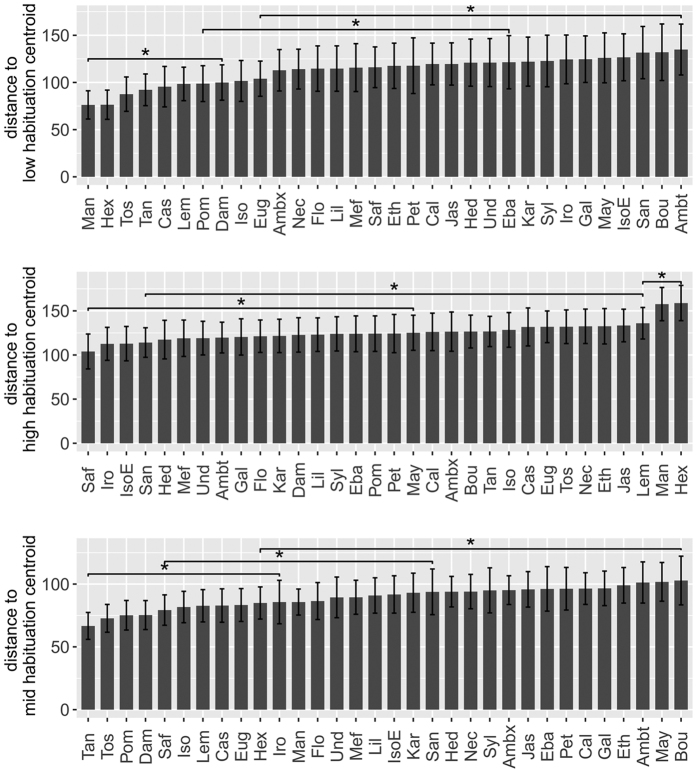
Differences of habituation between odorants. Habituation is measured as the mean city block distances (±CI95%) to each centroid curve a) low habituation, b) high habituation and c) mid habituation. There were numerous significant differences (Tukey-test *fdr* corrected) between odorants, 3 examples of the smallest significant differences are shown (*corresponds to p-adj < 0.05).

**Figure 3:**
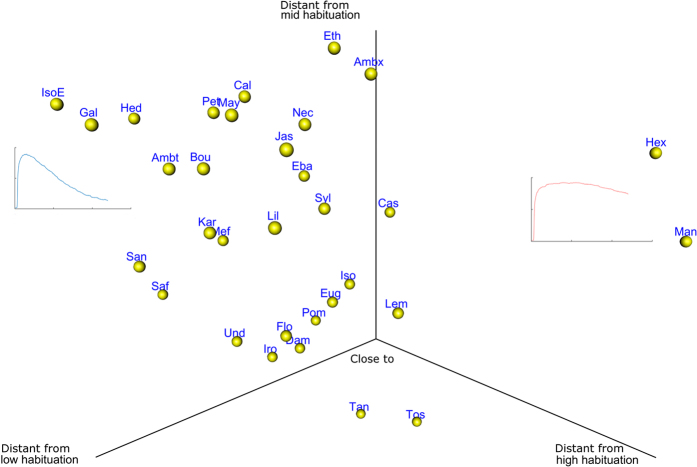
Distribution of the 32 odorants in a habituation space. X, y and z axis represent the distance to each centroid curve. One sphere represents one odorant which coordinates are the median distances to each centroid. The size of the spheres emphasizes the perspective. The blue and red curves represent the profile of habituation (intensity as a function of time) for the extreme odorants. Hex ad Man present profiles of habituation similar to the low habituation centroid while IsoE, Gal, San and Saf are more similar to the high habituation centroid (blue curve).

The mean distances to each centroid (high, mid and low habituation), representing a measure of similarity to each profile of habituation, were compared between odorants. Odorants differed in similarity to the centroid curves with low habituation (F[31, 1451] = 5.18, p < 0.0001), mid habituation (F[31, 1451] = 3.43, p < 0.0001) and high habituation (F[31, 1451] = 3.76, p < 0.0001) (Fig. 2). It appeared that the distance to low habituation enabled to better discriminate odorants, as there were more significant differences than with both other centroids (low habituation: 142 significant differences, mid habituation: 83 and high habituation: 77). With the mean distance to each centroid as coordinates we could represent the odorants in a habituation space going from a high to low habituation (Fig. 3). The odorants Man and Hex were clearly separated from other odorants and showed a low habituation profile with very few subjects presenting high habituation. On the other hand IsoE, Gal, Saf and Sand matched the high habituation profile.

### Physicochemical and sensory determinants of habituation

With 15 explanatory variables that were likely to present multiple collinearities, standard linear regression models failed, therefore data were analysed with partial least square regression (PLSr) method. This method allows to consider a high number of independent variables for a lower number of dependent variables, and to overcome collinearities within each block of variables.

#### Choice of dimensions

**Figure 4:**
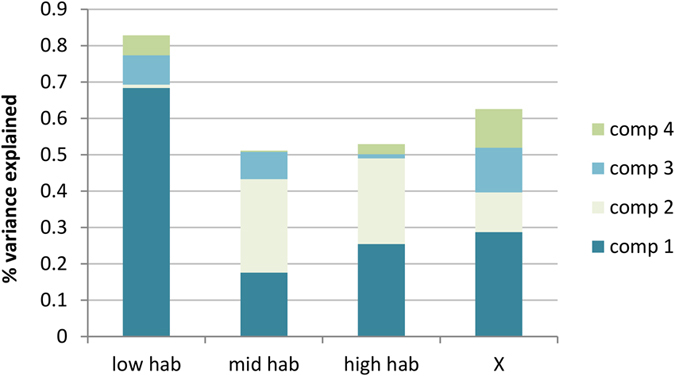
Percentage of variance explained by each of the 4 components selected. X bar corresponds to the % of variance used from the explanatory variables and lowhab, midhab and highhab bars correspond to the % of variance explained in each response variable. The variance explained is decomposed into the percentage explained by each component. The sum represents the total amount of variance explained by the 4 components together.

The leave-one-out validation method (Supplementary Figure 2) revealed that the lower root mean squared error of prediction occurred for 4 components for the lowhab variable and for 3 components for the midhab and highhab variables. The 4 first components explained 62.51% of the variance of X and 82.80%, 51.08% and 52.90% of the total lowhab, midhab and highhab variances respectively (Fig. 4). Lowhab is mostly explained by the 1^st^ component, while for midhab and highhab, the 3rd and the 4^th^ together explained most of the variance. Therefore in the following analysis, the first four components were considered.

#### Physicochemical and sensory explanatory variables

The composition of the components included sensory as well as physicochemical variables. The composition will be detailed in terms of loading. weights (lw), which can be understood in a similar fashion to correlations, in the following result section. The *vip* value will be given as a result of the importance of the variable in the projection. It allows to order the importance of each variable. All the result should be considered relatively to the % of variance explained. In the following section the results are presented for the habituation variables that best separated the odorants, namely low hab and high hab (Fig. 3) for which 82.80% and 52.90% of the variance is explained by the 4 components (Fig. 4). A summary of the significant effects of each variable on habituation is presented in Table 2.

**Figure 5:**
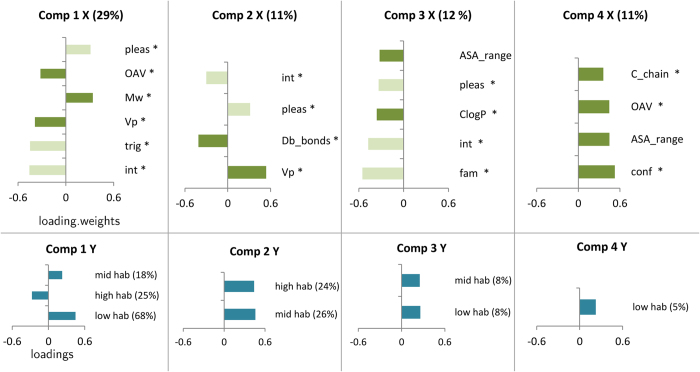
The linear combinations (loadings and loading.weights respectively) of X (explanatory) and Y (response) variables are presented for each component. Only variables that presented loadings or loading.weights >0.3 were represented. The contribution of each variable to the component (measured by variable of importance for the projection: VIP > 1) is highlighted by (*). The percentage of variance explained is represented in brackets. Comp = component, hab = habituation, pleas = pleasantness, int = intensity, fam = familiarity, Vp = vapour pressure, Mw = molecular weight, OAV = odor activity value, Db_bonds = double bonds, C_chain = carbon chain length, conf = number of conformers, logP = hydrophobicity, ASA_range = accessible surface area.

The first component explained 68% of the distance to low hab centroid (l = 0.45) which negatively correlated with intensity (lw = −0.45, vip = 2.63), trigeminality (lw = −0.44, vip = 2.58), Vp (lw = −0.38, vip = 2.23), and OAV (lw = −0.32, vip = 1.83) (Fig. 5). Therefore the odorants that produced low habituation were more intense, more trigeminal, had higher Vp and higher OAV. The variance of low hab regressed as the first component was negatively correlated with Mw (lw = 0.34, vip = 1.95) and pleasantness (lw = 0.31, vip = 1.78) (Fig. 5). Thus, the odorants with low habituation had also a lower Mw and a lower pleasantness. The first component was on the converse positively correlated to the distance to high hab (l = −0.27) and explained 25% of the variance (Fig. 5). Hence, the odorants that were similar to the high hab centroid had the same predictors but with opposite effects, although this prediction may not be as accurate as those for distances to low hab centroid.

The second component explained 24% of the variance of the distance to high hab centroid. High hab variable correlated positively with the 2^nd^ component (l = 0.46) and therefore negatively with the number of double bonds (lw = −0.41, vip = 1.65) (Fig. 5). Odorants with a high habituation had more double bonds.

The third component explained 8% of the variance of the distance to low hab centroid. Low hab variable correlated positively with the 3^rd^ component (l = 0.26) and therefore negatively with familiarity (lw = −0.55, vip = 1.13) and clogP (lw = −0.36, vip = 1.01) (Fig. 5). Odorants with low habituation were potentially more familiar, and were more hydrophobic.

Finally the 4^th^ component explained 5% of the variance of the low hab variable. This variable correlated positively with the 4^th^ component (l = 0.22). This 4^th^ component, although it explains only a small part of habituation variables, brings essentially physicochemical predictors. Indeed the number of conformers correlated positively with low hab (lw = 0.52, vip = 1.10), as well as the length of carbon in chain (lw = 0.36, vip = 1.12). Therefore, odorants with lower habituation potentially have less carbon in chain and less conformers.

## Discussion

In line with Rankin^11^ and Stone *et al*.^14^ we highlighted new principles of habituation by measuring habituation with a non-declarative method in a large set of 32 odorants varying in physico-chemical characteristics. Although habituation was relatively short (120 s), the molecular characteristics of the odorants appeared to have contributed to the differential filtering of the olfactory information. Indeed, the vapour pressure, the molecular weight, the Odor Activity Value (OAV) and the number of double bonds mainly contributed to modulate habituation. These physico-chemical characteristics may at least partly explain the importance of trigeminality in habituation. Trigeminality is highly correlated to intensity and pleasantness, which also contribute to the differential habituation. Moreover, although they explain only a small part of the variance in the habituation space, the length of the carbon chain, the number of conformers and the logP also contributed significantly to habituation.

The method used here, continuous recording of the intensity with the intensity-like pressure device and the Kmeans clustering analysis, resulted in the data being clustered as a function of habituation. The methodology appears as conclusive to study habituation. The partial least square regression (PLSr) model is validated by the high percentage of variance explained, more than 80% of the low habituation similarity and more than 50% of the high and mid habituation similarities. Furthermore, the model used more than 60% of the explanatory variables, among which structural and sensory variables. To further validate the model, the next step should be to predict the habituation of a new set of molecules and verify, with the same method as the one presented here, whether the observed habituation fits predictions. Although PLSr is a robust method to isolate predictors among collinear variables, one should keep in mind that the explanatory variables are not always independent^15^. For example, trigeminal molecules are usually perceived as more intense and less pleasant^16^,^17^. Therefore, in the next sections we will discuss each of these effects separately and in combination with other effects, as it is likely that some perceptual characteristics may be correlated and that some physico-chemical characteristics may explain the perceptual characteristics.

Trigeminality appeared to largely impact habituation, almost as much as intensity, which was already highlighted as the main factor modulating habituation^2^,^11^,^13^. The effect of trigeminality in psychophysical studies is often underestimated because they do not correspond to a unique category of sensations. Indeed, trigeminal nerves are composed of different somatosensory and pain fibers that can react to texture, temperature, or chemicals. Their description can be as variable as burning, fizzy, soft, warm, cold, tingling, prickling, pungent, creamy, irritating, etc. Therefore, psychophysical evaluation of the trigeminal sensations is laborious. “Trigeminality” here, refers exclusively to two main aspects of trigeminal sensations, prickling and warmth, due to technical constraints. However the effect of trigeminality appears clearly as a key factor in habituation. The effect of trigeminal system on olfactory perception has been clearly shown, notably in neurosciences. The trigeminal system can be seen as a sentinel of the body, present to prevent from breathing or eating noxious compounds. Trigeminal and olfactory systems are highly connected both at peripheral^18^,^19^ and central levels^20^. Piriform and orbitofrontal cortices have been shown to process both olfactory and trigeminal stimulation^19^,^20^. Interestingly, these two areas are also key areas for the control of habituation^10^. Finally, trigeminality can be seen as the sentinel of the olfacto-respiratory system, where trigeminal molecules may act as signal of threat, that will largely impact habituation.

We identified that habituation decreased with unpleasantness. This variable appeared on the 1^st^ component which explained 28% of the variance of the habituation variables. Conversely to our result, several studies showed that subjects habituated faster to malodor such as H_2_S or skatol which smell like “rotten eggs” and “feces”, respectively^21^,^22^,^23^. However none of our odorants were very unpleasant and would qualify as a “malodor”. Furthermore, these odorants were also more trigeminal which likely correlates with pleasantness^17^. On the other hand, very unpleasant and non-trigeminal odorants (4-methyl-pentanoic acid: cheese like odor) show high habituation^24^, in contrast to our trigeminal and slightly unpleasant odorants (hexenol and manzanate: alcoholic, fruity like odors) which induce low habituation. Furthermore, Flohr *et al*.^25^ showed that CO_2_, the only trigeminal stimulant, tends to increase habituation. Altogether these results suggest that only a combination of factors affect habituation, and not an effect of pleasantness or trigeminality alone.

We identified that odorants with a lower habituation, had a lower molecular weight, lower carbon chain, higher vapour pressure and higher AOV (activity odor value). AOV is directly linked to the vapour pressure (ratio between the detection threshold and the vapour pressure). Our results are in line with Stone *et al*.^14^ who found that odorants with lower habituation had a higher Vp. Indeed, a high Vp is associated with pungency^17^,^26^. Furthermore they are in accordance with Doty *et al*.^17^ study, which showed that pungency increases when odorants have lower molecular weight, lower vapour pressure and lower retention time. Similarly, Cometto-Muniz and Cain^26^ found in series of ketones, alcohols and acetates, that pungency increased in low molecular weight, high vapour pressure and smaller carbon chain odorants. Therefore, a low molecular weight, a small carbon chain and a high vapour pressure may at least partly explain the higher trigeminality of the odorants and the resulting lower habituation.

In our study, reduced number of double bonds partly explained higher habituation. The number of double bonds is a factor involved in the binding of the ligand to olfactory receptors, OR^27^,^28^,^29^,^30^,^31^,^32^,^33^,^34^. A slight change in the number of double bonds has large repercussions on receptor interaction. Araneda *et al*.^27^ showed that rat I7 OR are less activated by molecules with higher number of double bonds. In the human OR17-40, changing a single bond into a double bond transforms agonist ligand into antagonist^30^. At the level of the olfactory bulb, the number of double bonds appears as a decisive factor for the odotopy of odorants^28^,^29^; which may explain why adding a single double bond has striking changes on perception. For example, Linalool and dihydrolinalool have similar refreshing floral, woody, and citrus odors, but when adding a double bond, linalool smells like mushroom and dihydrolinalool as sweet, herbaceous and nutty^32^.

The three variables, “hydrophobicity”, “number of conformers” and “carbon chain length” have an effect on habituation, although to a lesser extent than other physicochemical variables exposed above. In the literature, these molecular characteristics regularly appear as key factors in structure-activity studies. The odorants that induced lower habituation were more hydrophobic. Hydrophobic proteins hardly pass the mucus barrier of the olfactory epithelium, and have to be transported to the receptors by olfactory binding proteins (OBP)^35^. This differential pathway through the mucus may impact the connection with olfactory receptors, which may convey different information to the brain and thus impact habituation. The carbon chain length consistently appears as a determining factor in the structure-activity relationship^27^,^31^,^36^,^37^,^38^,^39^. Furthermore, a smaller carbon chain is consistent with a lower Mw, found to impact substantially habituation. Finally, the number of conformers is a concept which receives little attention in structure-activity relationships. However, it may be that the number of conformations a molecule can adopt is a strong determinant of the number of receptors the molecule will be able to activate.

Habituation exists in all sensory systems. For example, habituation in vision can be observed as a typical after-images effect^40^. When fixing a black square with a white dot for a minute, and then looking at a blank white page, the negative image appears. Because the neurons need some time to recover from adaptation, this effect highlights the adaptation of visual neurons to the features of the image. Our results are in line with results in other sensory modalities. For example, visual or auditory habituations appeared to differ between different stimulations. It was found that habituation to a tiny flash or gratings is modulated by the size and spatial frequency of the flash but not by the orientation of gratings^41^. Concerning audition it was also found that the degree of habituation was varying among frequencies, as shown in gerbil^42^.

In conclusion, we found that habituation decreased when odorants had more double bonds, smaller molecular weight, higher vapour pressure, and to a lesser extent lower hydrophobicity, smaller carbon chain and less conformers. Additionally, these structural characteristics have scope to lead to differential sensory properties such as higher intensity, higher trigeminality and lower pleasantness. All together our results suggest that differential habituation to odorants depend on combinations of factors. Therefore, taking in account multiple variables appear as a sound method to address structure-activity issues in olfaction. Furthermore, the method of measurements as developed in this paper is of interest for the measure of habituation. On the contrary to classical declarative methods of sensory evaluation, (e.g. visual analog scale), the intensity-like pressure device indeed takes into account the variability of intensity more than the value of intensity on its own. It is less cognitively demanding than visual analog scale. Although we did not directly measure the method reliability from day to day, we do have results of a second study on 6 of the odorants used here, rated by 30 subjects, with the exact same materials and methods. We found very similar results of habituation to each odorant, suggesting that the measure of habituation is reliable. Finally, these results have been found in young healthy adults but should be investigated in other age population, or in populations with different health conditions, in order to pinpoint variation of habituation due to differential neuronal connectivity and/or receptors functionality and distribution. These results may also be of interest for the understanding of targeted inhibitory mechanisms by the central nervous system and the receptor-ligand activity. Indeed, if receptors or neuronal activity are heterogeneously inhibited or adapted, then it means that studying habituation or adaptation to different molecules may enable us to gain insights in the receptor or neuronal topography.

## Material and Methods

### Subjects

Fifty-one participants (26±5 y.o.; 33 women and 25 men) were recruited. They were healthy and had a normal sense of smell as verified with “Sniffing’ Sticks”^43^. The entire procedure was explained before subjects entered the study and was again explicitly detailed orally and on paper during the first meeting. Subjects then signed an informed consent form. The study was conducted according to the Declaration of Helsinki and was approved by the Ethics Committee of the Technical University of Dresden Medical School (EK13012013).

### Odorants

Thirty two odorants (Givaudan Uk Ltd, Ashford, UK) were selected for their extreme values in physicochemical characteristics (Table 1). The physicochemical characteristics considered were (**1**) the Accessible Surface Area (ASA_range), which represents the inward-facing surface part of the van der Walls surface (“reentrant surface”). (**2**) The number of conformers was also used as a variable; it indicates the number of different positions the molecules may adopt. It was calculated by means of DS 3.5, Accelrys-Biovia (“Best” conformer generation of maximum 255 conformers in energy range 20 kcal mol^−1^). (**3**) The number of carbons in the carbon chain of a molecule (C_chain), (**4**) the number of double bonds (Db_bonds), (**5**) the molecular weight (Mw), (**6**) the chemical family (Cfamily) and (**7**) the hydrophobicity partition coefficient of the molecules (logP) were taken in account. (**8**) The saturated Vapour pressure (Vp) is an indicator of the liquid’s evaporation rate. (**9**) The Odor Activity Value (OAV) corresponds to the ratio between the concentration of an odorant in a sample and the detection threshold of the odorant. Odorants were selected for their extreme values regarding ASA_range, C-chain, logP and Db_bonds. We selected 4 odorants with high values and 4 odorants with low values in each of these 4 physico-chemical properties. These properties were chosen because they are commonly used in structure activity studies^27^,^31^,^35^,^36^,^37^,^38^,^39^. (**10**) The classification in the olfactory families was also considered (Ofamily) as well as (**11**) the session in which each odorant was presented to subject (Pres_order). Moreover, **4 sensory characteristics** (intensity, pleasantness, familiarity, trigeminality) were directly measured by the participants during the test sessions. In the end, 15 variables were considered to characterize the odorants.

Odorants were diluted using Propylene glycol (PG, Sigma Aldrich) in order to establish iso-intense odorants (Supplementary Table 1). Diluted odorants (25 ml) were filled into custom made “washing bottles” (total volume 250 ml) and delivered with a mobile olfactometer^44^. The solutions were replaced every 15^th^ subjects in order to maintain constant concentrations. The solutions were also regularly smelled by the experimenter, in order to check for noticeable change of concentration. The airflow was set to 7.3 L/min and divided equally between two bottles, a control bottle filled with water (25 ml) and the bottle with the odorant. Therefore, even without sniffing, the humidified and odorized airflow could reach the olfactory epithelium. Air flow was controlled before each subject started the experiment.

### Experimental Procedure

Before each session subjects evaluated the intensity, pleasantness, familiarity and trigeminal sensation evoked by the odor. Subjects described the odorants on printed scales with 9 levels (1 was associated with terms “low”, “unpleasant”, “unfamiliar” or “not prickling or not warm”; and level 9 with “strong”, “pleasant”, “familiar” or “prickling or warm”, respectively). Coldness or freshness of trigeminal stimuli was not evaluated as odorants were presented at room temperature and not body temperature which would have biased this measure.

Before starting the experiment, subjects were trained to breathe regularly through the mouth and to not sniff during 120 s. Accuracy of the breathing was checked by a custom-built anemometer placed directly in front of the nostrils. This technique was used in order to limit dishabituation that could arise when subject sniffs. The odorant was delivered continuously, via Teflon tubing to the subject’s nose, during 120 s at a constant concentration. Between odorants, an inter-stimulus interval of 90 s was observed in order to recover from habituation.

**Figure 6:**
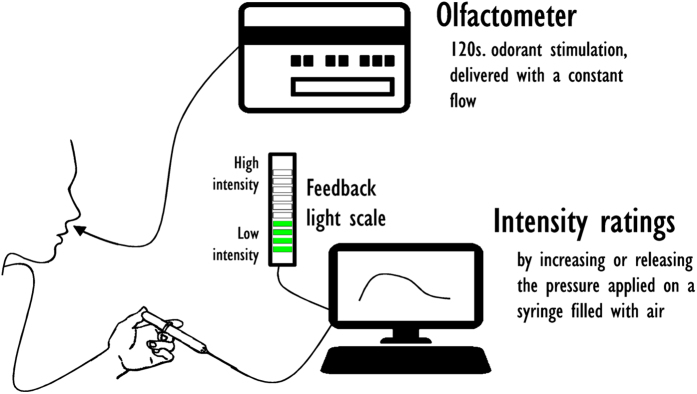
Design of the method. The subject is constantly stimulated with odorized air, at a constant flow, during 120 s (olfactometer). Simultaneously, they evaluated intensity, by increasing or releasing the pressure applied on a syringe filled with air (intensity-like pressure device). The pressure applied is transformed by and AD converter into a value of intensity between 0 and 100. Subjects received a feedback of their continuous rating on a light scale. The intensity-like pressure device is calibrated at the beginning of each day of testing. (*Computer image has been designed by lvaro_cabrera - Freepik.com. Olfactometer image has been designed by Freepik. Hand and syringe image has been designed by johnny_automatic from vector.me.*).

Subjects had to evaluate continuously the intensity of the odorant using pressure as a measure of odor intensity (intensity-like pressure device, Fig. 6). The device, specifically developed for this study, was composed of a syringe filled with air and connected to an A/D converter, which transforms the pressure applied into a value between 0 and 100, which corresponds to the intensity perceived by the subjects at a time t. The frequency of recording was 4 Hz (1/250 ms). The recordings thus consisted of 481 data points for each odor and each subject. Subjects received feedback on the pressure applied to the syringe from a scale composed of 10 lights. The syringe filled with air was calibrated each day and checked before each subject used it to ensure a reliable measure of odor intensity. As we were interested in variation of intensity occurring during the 120 s and as the first data point (intensity when the odorant is detected) may have conditioned the range of value the subject could use, subjects were asked to start evaluating intensity at the 6th level, out of 10, on the visual scale. Then, subjects could modulate the pressure applied (relaxing or compressing the air) as their perception of intensity would change. The 32 odorants were tested in 4 sessions. Within each session, odorants were chosen so that they would match as closely as possible in intensity; and their order of presentation was counterbalanced between subjects according to a latin square.

### Statistical analysis

All statistical analysis were performed using the free software R 3.3.0 (http://cran.r-project.org/) and Matlab (MATLAB, R2015b).

#### Dataset

Three subjects did not attend all sessions and were thus not included in the analysis. 5 subjects presented chaotic data, with high frequency change of intensity ratings, supposedly following breathing, and were therefore not included in the analysis. Furthermore, 13 subjects presented a delay of response larger than 17 s (14% of the total habituation time) for 1 to 9 odorants. Therefore these ratings were not included in the analysis. Finally 229 curves were excluded and 1531 curves were analyzed. The curves were normalized on the intensity axis to avoid differences of intensity between odorants before habituation.

#### Clustering analysis

As curves presented a variety of shapes and as no *a priori* biologically relevant parameters could be extracted, data were analysed with a no *a priori* method, using k-means clustering (MATLAB, R2015b). When 3 clusters were chosen the silhouette analysis revealed the most homogeneous groups of data, compared with other number of clusters. Although the silhouette analysis showed that some data could belong to several clusters (Supplementary Information, Fig. 1), the 3 centroids were meaningful in terms of habituation (cf result section). They were identified as presenting a high habituation (high hab), a low habituation (low hab) and a middle habituation (mid hab). As the separation between the clusters was not very clear, we decided not to work directly with the clusters, but we worked on the distance of each curve to the three centroids, preserving in this way all the variability.

#### Differences of habituation between odorants

To test the difference of habituation between odorants we used Linear Mixed models fitted by maximum likelihood, with *subjects* as a random factor (“lme” function from “nlme” package in R). We tested three dependent factors which consisted in the city block distances of the data to each centroid curve (high hab, low hab and mid hab) and the odorants composed the independent factor. When the lme model was significant, we ran Tukey multiple comparisons, false discovery rate (fdr) corrected, to highlight differences between odorants. Alpha risk error was settled at p-corrected <0.05.

#### Physicochemical and sensory determinants of habituation

With 15 independent variables that likely presented multiple collinearities, standard linear regression models failed, therefore we analysed the data with partial least square regression (PLSr) method. The main R package used for multivariate data analyses was «pls 2.5-0»^45^. This method allows to consider a high number of independent variables for a lower number of dependent variables; and to overcome collinearity of certain variables. The Y matrix consisted of 3 response variables: the medians of distances to the three centroid curves by odorants (Y matrix: Lowhab, Midhab and Highhab). This particular way to calculate the response variables signify that the higher the value of the response variable, the higher the distance to the centroid is, and the lower is its similarity to the corresponding habituation profile. Thus, in a context of PLSr analysis, one Y response with a high loading.weight on a component indicates a low similarity to the habituation class on this component and conversely. The X matrix consisted in the physicochemical and sensory characteristics of each odorant. The categorical variables (Cfamily [7 levels], Ofamily [10 levels] and Pres_order [4 levels]) had to be presented in a disjunctive table, as PLS does not accept categorical variables. The X matrix contained 33 variables. X and Y were mean-centred and normalized. The levels of each categorical variable were rescaled according to the total number of levels in each variable (each level was divided by the total number of levels in the variable), to avoid overweighed contribution of individual levels to the PLS. The PLSr model was fitted with the default set method “kernel” and was cross-validated with the “leave-one-out” method.

Root Mean Squared Error of Prediction (RMSEP) were calculated for each possible component (2 to 32) in order to find the best number of components in X that explain the maximum of variance in Y (Supplementary Information, Fig. 2). The loading.weights of the X variables used to compose the components were reported in order to interpret the components, as well as the loadings of Y. In order to facilitate interpretation, only variables that had loading.weights >0.3 were considered. Concerning the Y variables, only those with a loading >0.2 were considered.

Finally the relative importance of the X variables was assessed with the variable importance in the projection method (“vip” function, adapted from *mixOmics* package in order to accept *plsr* results). Only VIPs >1 were considered as contributing significantly in the projection^46^.

## Additional Information

**How to cite this article**: Sinding, C. *et al*. New determinants of olfactory habituation. *Sci. Rep.* **7**, 41047; doi: 10.1038/srep41047 (2017).

**Publisher's note:** Springer Nature remains neutral with regard to jurisdictional claims in published maps and institutional affiliations.

## Supplementary Material

Supplementary Information

## Figures and Tables

**Table 1:** Description of the odorants selected for the study and their physicochemical characteristics.

CAS#	Abb.	Name	ASA	C_chain	logP	Db_bd	Conf.	Mw	Vp	Ofamily	Cfamily
55066-48-3	Mef	MEFROSOL	**42.20**	5	2.7	3	17	178	0.0004	Floral	ALCO
676532-44-8	Syl	SYLKOLIDE	**86.71**	5	4.4	2	144	268	0.0046	Musky	ETHER
81782-77-6	Und	UNDECAVERTOL	**63.17**	10	3.9	1	64	170	0.0110	Green	ALCO
65113-99-7	Sand	SANDALORE	**38.60**	5	4.73	1	106	210	0.0010	Woody	ALCO
488-10-8	Jas	JASMONE	**16.19**	5	2.8	3	6	164	0.0133	Floral	KETO
97-54-1	Iso	ISOEUGENOL	**8.34**	3	2.1	4	23	164	0.0040	Spicy	ETHER
16510-27-3	Tos	TOSCANOL	**11.09**	3	3.8	3	1	162	0.0113	Agrestic	ALCO
54440-17-4	Saf	SAFRALEINE	**0**	1	2.9	4	7	174	0.0120	Spicy	KETO
928-96-1	Hex	HEXENOL Cis-3	20.20	**6**	1	1	37	100	0.5333	Green	ALCO
80-54-6	Lil	LILIAL	14.19	**3**	4.2	4	15	204	0.0040	Floral	ALDE
67801-20-1	Eba	EBANOL	24.20	**5**	4.2	2	39	208	0.0054	Woody	ALCO
873888-84-7	Kar	KARMAFLOR	60.71	**6**	5	5	83	234	0.0010	Floral	ESTER
28940-11-6	Cal	CALONE	4.89	**1**	1.2	4	70	178	0.0133	Aldehydic	ETHER
95962-14-4	Nec	NECTARYL	39.30	**1**	4.8	2	4	220	0.0001	Fruity	KETO
28645-51-4	Ambt	AMBRETTOLIDE	44.47	**0**	6	2	78	252	0.0003	Musky	LACTONE
916887-53-1	Pet	PETALIA	9.69	**1**	3.45	4	46	211	0.0002	Floral	NITRILE
6790-58-5	Ambx	AMBROFIX	0.00	1	**6**	0	11	236	0.0013	Woody	ETHER
871465-49-5	Cas	CASSYRANE	11.67	3	**4.45**	1	21	182	0.1040	Fruity	ETHER
79-69-6	Iro	IRONE ALPHA	14.44	4	**3.8**	3	3	206	0.0053	Floral	KETO
54464-57-2	IsoE	ISO E SUPER	9.59	2	**5.7**	2	15	234	0.0027	Woody	KETO
166090-45-5											
97-53-0	Eug	EUGENOL	7.54	3	**2**	4	27	164	0.0112	Spicy	ETHER
08.11.4940	Eth	ETHYL MALTOL	6.70	2	**1.69**	3	22	140	0.1333	Sweet	ETHER
125109-85-5	Flo	FLORHYDRAL	18.20	3	**3.1**	4	5	190	0.0065	Aldehydic	ALDE
32669-00-4	Tan	TANAISONE	13.93	2	**3**	2	34	152	0.0970	Agrestic	KETO
1222-05-5	Gal	GALAXOLIDE	3.28	1	5.9	**3**	18	258	0.0133	Musky	ETHER
18127-01-0	Bou	BOURGEONAL	14.99	1	3.2	**4**	40	190	0.4037	Floral	ALDE
57378-68-4	Dam	DAMASCONE δ	15.76	4	4.2	**3**	5	192	0.0267	Floral	KETO
357650-26-1	Pom	POMAROSE	14.50	8	3.3	**4**	10	166	0.1410	Fruity	KETO
39255-32-8	Man	MANZANATE	37.91	4	2.65	**1**	17	144	1.9997	Fruity	ESTER
61792-11-8	Lem	LEMONILE	34.64	2	3.1	**2**	83	163	0.0170	Citrus	NITRILE
0013828-37-0	May	MAYOL	20.49	2	3.24	**0**	141	156	0.0053	Floral	ALCO
24851-98-7	Hed	HEDIONE	63.87	5	2.9	**2**	56	226	0.0013	Floral	ESTER

Odorants were chosen according to four physico-chemical characteristics (ASA, C-chain, logP, Db_bd). In each category, four odorants with a high value and four odorants with low values were chosen (cells with bold font). Cas# = chemical abstract service (identification of the molecule), Abb. = abbreviation used in the manuscript, ASA = Accessible surface area, C_chain = number of carbon in chain, logP = hydrophobicity, Db_bd = number of double bonds, Conf. = number of conformers, Mw = molecular weight, Vp = vapour pressure, Ofamily = olfactory family and Cfamily = chemical family. OAV (odor activity value) was also a variable but the data are not shown here, for confidentiality purposes.

**Table 2:** Sensory and physicochemical determinants of habituation.

	int	trig	Vp	Mw	OAV	pleas	Db_bd	fam	C_chain	conf	logP
Less habituation when	↗	↗	↗	↘	↗	↘	↘	↗	↘	↘	↗
VIP	2.63	2.58	2.41	1.95	1.83	1.8	1.63	1.13	1.12	1.1	1.01
component	1	1	2	1	1	1	2	3	4	4	3

Low hab = low habituation, Mid hab = mid habituation and High hab = high habituation. Vp = vapour pressure, Mw = molecular weight, OAV = odor activity value, Db_bd = double bonds, C_chain = carbon chain length, conf = number of conformers, logP = hydrophobicity. The variables have been ordered relatively to the VIP values (>1), from the more important on the left to the less important on the right.
