# Supplementary material for: New determinants of olfactory habituation
Source: Sci Rep. 2017 Jan 25;7:41047. doi: 10.1038/srep41047 (PMC5264389; doi:10.1038/srep41047)
Supplement: Supplementary Information [file srep41047-s1.pdf]

Supplementary Information to:

“New determinants of olfactory habituation”

Charlotte Sinding[1,2]*, François Valadier[3], Viviana Al-Hassani[1], Gilles Ferron[2], Anne Tromelin[2], Ioannis Kontaris[4], Thomas Hummel[1]

1: Smell & Taste Clinic, Department of Otorhinolaryngology, University of Dresden Medical School, Dresden, Germany.

2: Centre des Sciences du Goût et de l’Alimentation, CNRS, UMR 6265, INRA, UMR 1324, Université de Bourgogne, Dijon, France

3: Open Value, Paris, France

4: Givaudan UK Ltd, Ashford, UK

Corresponding author:

*Dr. Charlotte Sinding,

Centre des Sciences du Goût et de l'Alimentation, INRA UMR 1324, 17 rue Sully, 21000 Dijon, France

Tel : +33 380 69 33 66

csinding@dijon.inra.fr

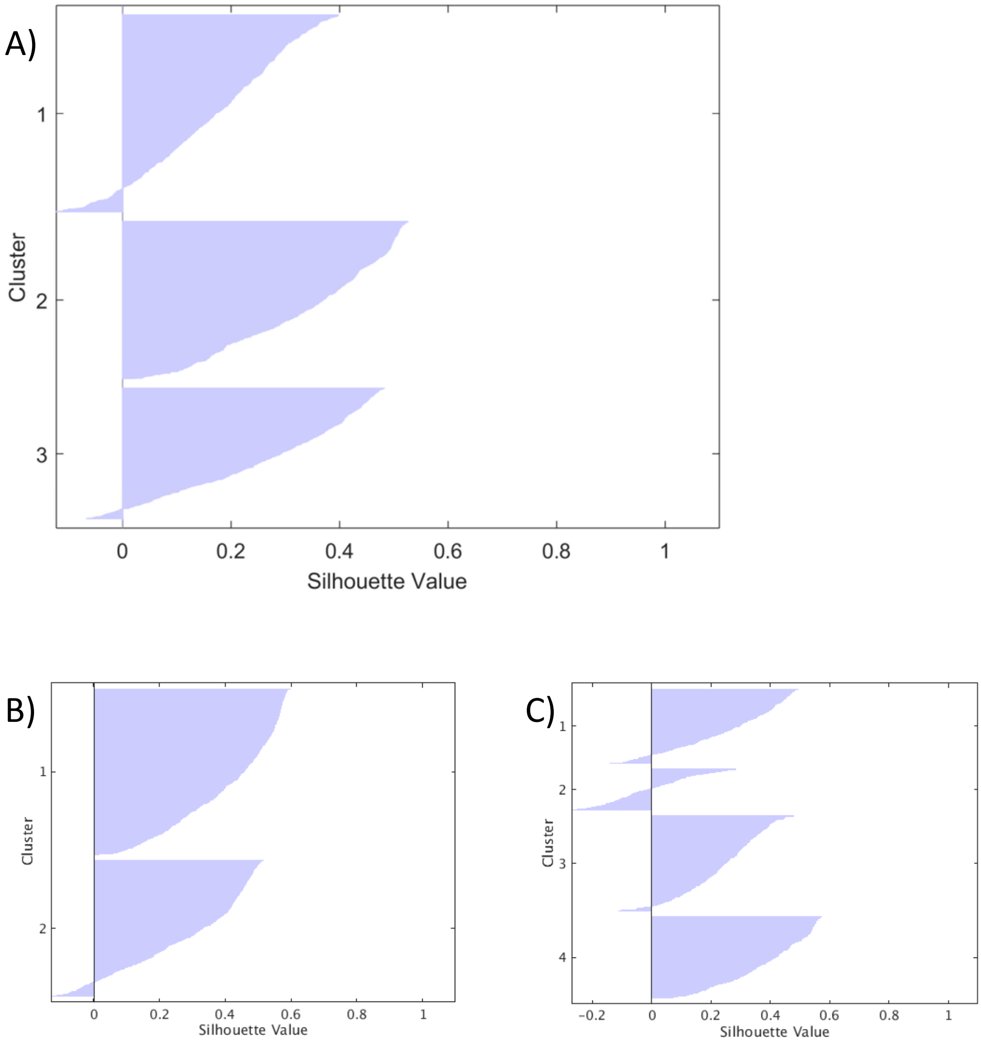

Supplementary Figure 1: Silhouette Analysis: Representation of the homogeneity of data within each cluster and separation from other clusters (silhouette values). Each data is represented by one line which all together forms a silhouette. In each cluster data are ordered by how close they are from the centroid curve and how far they are from the two other centroid curves. The silhouette value is a measure of how similar a curve is to its own cluster (cohesion) compared to other clusters (separation). The silhouette range from -1 to 1; where a high value indicates that the curve is well matched to its own cluster and badly matched to neighboring clusters. The more the silhouette has a rectangular shape the better is the cohesion and separation. In the silhouette analysis with 3 clusters (A), which is the best fit compared with 2 (B) and 4 clusters (C), the cluster 2 had the centroid “low habituation”, which had the best silhouette compared with the other clusters. The cluster 3 (high habituation) also had a good fit but some silhouette values were negative and the data could therefore belong to another cluster. The first cluster (mid habituation) presented lower silhouette value and contained more data with negative silhouette values, which revealed that this last cluster did not separate the data very well. The silhouette analysis with 2 clusters (B) contains lot of data with a low silhouette value in the cluster 1. The silhouette analysis with 4 clusters (C) showed that the second cluster is not cohesive and well separated from others.

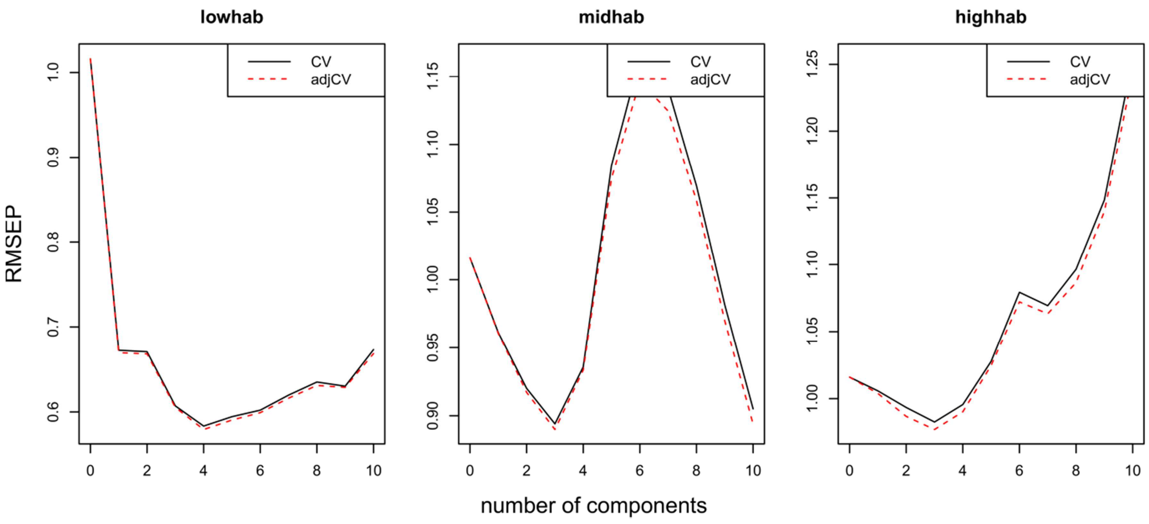

Supplementary Figure 2: **Root mean squared error of prediction (**RMSEP), cross-validation estimate, as well as the adj-CV, which is adjusted for bias. RMSEP are presented for each variable of habituation. The lowest RMSEP highlights the number of components which best explain the data. For the distance to low habituation centroid the data are best explained with 4 components, while for the 2 other response variables 3 is the best fit.

Supplementary Table 1: Concentration in Propylene Glycol of each molecule (% of dilution (w/w)), and repartition in the 4 sessions of testing. The last session was conducted together with mixtures of odorants which are not reported here, that is the reason one only one odorant is reported in session 4.

	**odorant**	**ratio**		**odorant**	**ratio**		**odorant**	**ratio**
	Manzanate	1.7%		Lilial	10.0%		Damascone	3.3%
	Jasmone	10.0%		Mayol	5.0%		Cassyrane	2.0%
	Lemonile	2.5%		Ebanol	5.0%		Irone Alpha	2.5%
	Calone	2.0%		Hedione	5.0%		Florhydral	5.0%
	Eugenol	10.0%		Mefrosol	5.0%		Isoeugenol	7.5%
session 1	Nectaryl	4.5%	session 2	Ambrettolide	5.0%	session3	Pomarose	2.5%
	Ambrofix	4.0%		Undecavertol	5.0%		Toscanol	2.2%
	Hexenol	6.7%		Bourgeonal	6.7%		Safraleine	3.3%
	Ethyl maltol	2.7%		Karmaflor	4.0%		Iso E sup	6.7%
	Galaxolide	5.0%		Sylkolide	6.7%		Tanaisone	1.4%
							Sandalore	50.0%
						session 4	Petalia	2.5%
